# Delayed Serotonin Syndrome and Non-cardiogenic Pulmonary Edema Following Bupropion Overdose in a Seven-Year-Old Female: A Case Report and Review of Literature

**DOI:** 10.7759/cureus.56767

**Published:** 2024-03-23

**Authors:** Madison P Craft, Kaitlyn Burdsall, Hanna S Sahhar

**Affiliations:** 1 Pediatrics, Edward Via College of Osteopathic Medicine, Spartanburg, USA; 2 Pediatric Intensive Care Unit, Spartanburg Regional Healthcare System, Spartanburg, USA

**Keywords:** non-cardiogenic pulmonary edema, status epilepticus (se), prolonged qtc interval, serotonin toxicity, bupropion overdose

## Abstract

Bupropion is an atypical antidepressant prescribed for depression and attention-deficit/hyperactivity disorder and to aid in smoking cessation. Bupropion overdose management is largely aimed toward common sequelae, including seizures, tachycardia, and QTc prolongation. In this case report, we identify a rare event of pediatric bupropion overdose with aforementioned common sequela and atypical features, including a delayed presentation of serotonin syndrome and non-cardiogenic pulmonary edema. This case follows a seven-year-old Caucasian female with autism spectrum disorder (ASD) who presented in status epilepticus following an accidental bupropion overdose and required multiple anti-seizure medications, endotracheal intubation, and admission to the pediatric intensive care unit (PICU). The patient's condition improved, and she was extubated 25 hours after admission and transitioned to high-flow nasal cannula therapy. On day 3 of admission, she became febrile and developed dyspnea with decreased breath sounds and intercostal retractions, tachycardia, a rigid abdomen and extremities with sporadic tremors, pulmonary edema, and a prolonged QTc interval. Targeted therapies were initiated, and following treatment, our patient showed remarkable improvement in the subsequent 24 hours and was discharged home five days after the initial presentation. This case identifies a delayed presentation of uncommon and serious complications of bupropion overdose, including pulmonary edema and serotonin syndrome, in a pediatric patient. Prompt investigation and identification of bupropion toxicity can help practitioners mitigate further complications during admission and reduce morbidity and mortality.

## Introduction

Bupropion is an atypical antidepressant drug whose mechanism of action is not fully elucidated but is thought to inhibit norepinephrine and dopamine reuptake. It is a monocyclic aminoketone structurally similar to amphetamine [1]. Bupropion is indicated in the treatment of major depressive disorder and as an adjunctive therapy for smoking cessation. Typical side effects include nausea, insomnia, and nervousness, and it is contraindicated in patients with a history of seizures or eating disorders [2]. Bupropion is available in three formulations: immediate, sustained, and extended-release at varying doses [1,3,4].

Bupropion overdoses can present with seizures, agitation, tachycardia, hypotension, electrocardiographic abnormalities, nausea/vomiting, and cardiogenic shock [2]. Serotonin syndrome has been implicated in bupropion overdoses, although often involving concomitant selective serotonin or norepinephrine reuptake inhibitor (SSRI/SNRI) overdose [5]. Toxicity concerns with bupropion overconsumption are largely focused on seizure activity seen with as little ingestion as 600 mg [2]. The first steps in managing patients with suspected bupropion toxicity are maintaining the airway, breathing, and circulation to stabilize the patient, particularly if seizure activity is present. Some evidence postulates bupropion may block rapid delayed rectifier channels, which may cause QTc prolongation following overdose [6]. Possible cardiac conduction abnormalities should be assessed with a 12-lead electrocardiogram.

Treatment is mainly supportive for previously mentioned manifestations of bupropion overdose, with activated charcoal as an appropriate option if the patient has a secured airway and presents soon after known ingestion. Frequent vitals, point-of-care glucose, and bupropion blood level concentrations should be obtained, if available. Seizure occurrence varies with 17-41% of bupropion overdose cases; extended-release bupropion is implicated in seizures at higher levels than immediate- or sustained-release [2,7]. Status epilepticus has been described in prior case reports following massive bupropion overdoses in adults and children and in the death of pediatric patients in intentional overdoses [8-10]. Seizures should be managed per standard of care with benzodiazepines as first-line agents and further intervention for refractory seizures. Intensive care unit (ICU) settings should be utilized in patients with hemodynamic instability, refractory seizures, and life-threatening arrhythmias [2,11].

## Case presentation

A seven-year-old Caucasian female with a past medical history of autism spectrum disorder (ASD) presented to the emergency department (ED) with generalized tonic-clonic seizures following ingestion of an unknown substance and quantity. The patient's parents reported vomiting at home, followed by seizure-like activity and a sticky substance in her mouth.

**Figure 1:**
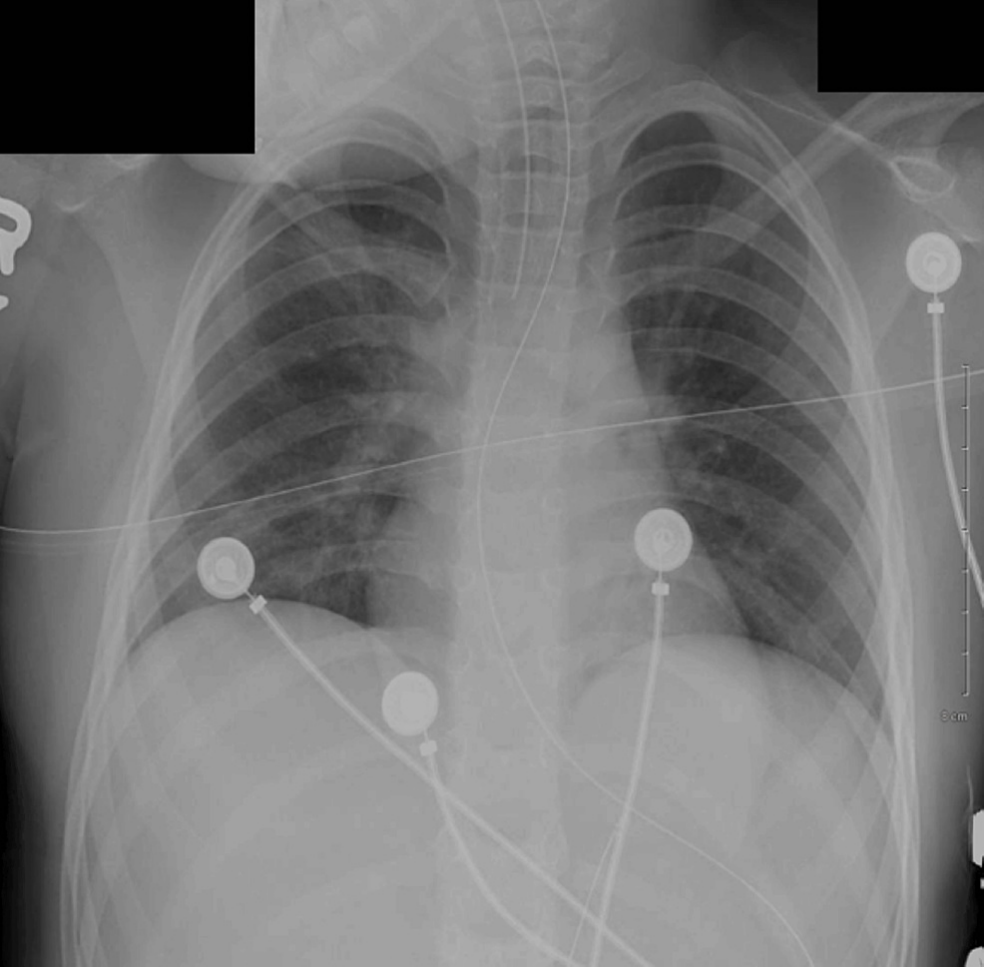
The chest X-ray taken after ET placement upon admission. ET: endotracheal tube

In the ED, three seizures occurred without a lucid period, with the third seizure occurring after lorazepam was given. A dose of midazolam was given followed by intravenous (IV) levetiracetam given when seizures had not ceased. Tracheal intubation following rapid sequence intubation protocol was initiated to protect the patient's airway. Post-procedural chest X-rays confirmed correct placement, and no cardiopulmonary abnormalities were noted, as shown in Figure 1. Propofol and midazolam IV drips were started for sedation post-intubation, and seizure activity ceased shortly thereafter. The patient's vital signs post-intubation were a blood pressure (BP) of 102/63 mmHg, heart rate (HR) of 131 beats per minute (BPM), respiratory rate (RR) of 18 respirations per minute (RPM), temperature of 99.7°F, and oxygen saturation (SpO2) of 100%.

**Figure 2:**
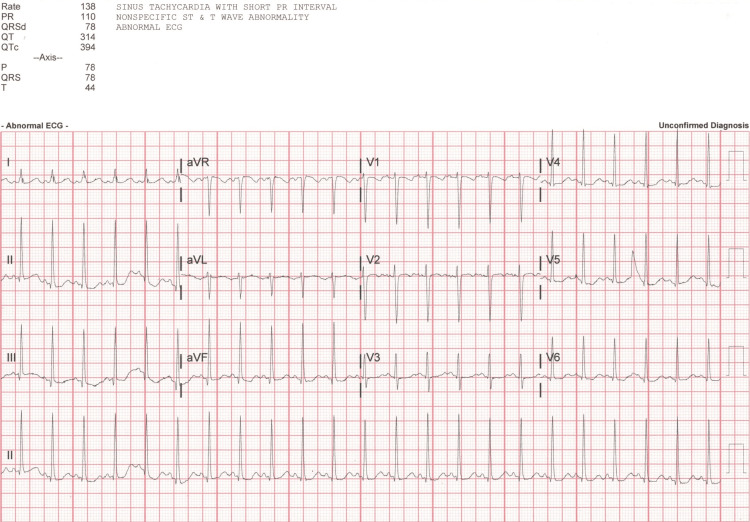
The patient's EKG at admission. EKG: electrocardiogram

The patient's ethanol, acetaminophen, and salicylate lab values were normal. A urine drug screen was positive for both benzodiazepines and amphetamines; however, it was collected post-benzodiazepine administration in the ED, and the child was not taking amphetamines for her ASD treatment. A computed tomography head without contrast showed no acute intracranial abnormalities, and an electrocardiogram (EKG) showed sinus tachycardia with a shortened PR interval and non-specific ST and T wave abnormalities, as shown in Figure 2. The patient was admitted to the pediatric ICU (PICU) for further management.

Following admission to the PICU, a 24-hour continuous electroencephalogram (EEG) showed no focal slowing or epileptiform activity and was interpreted as a normal sleeping EEG. Capillary blood gasses were ordered every six hours for respiratory and oxygen status assessment and were within normal limits during days 1 and 2 of admission.

**Figure 3:**
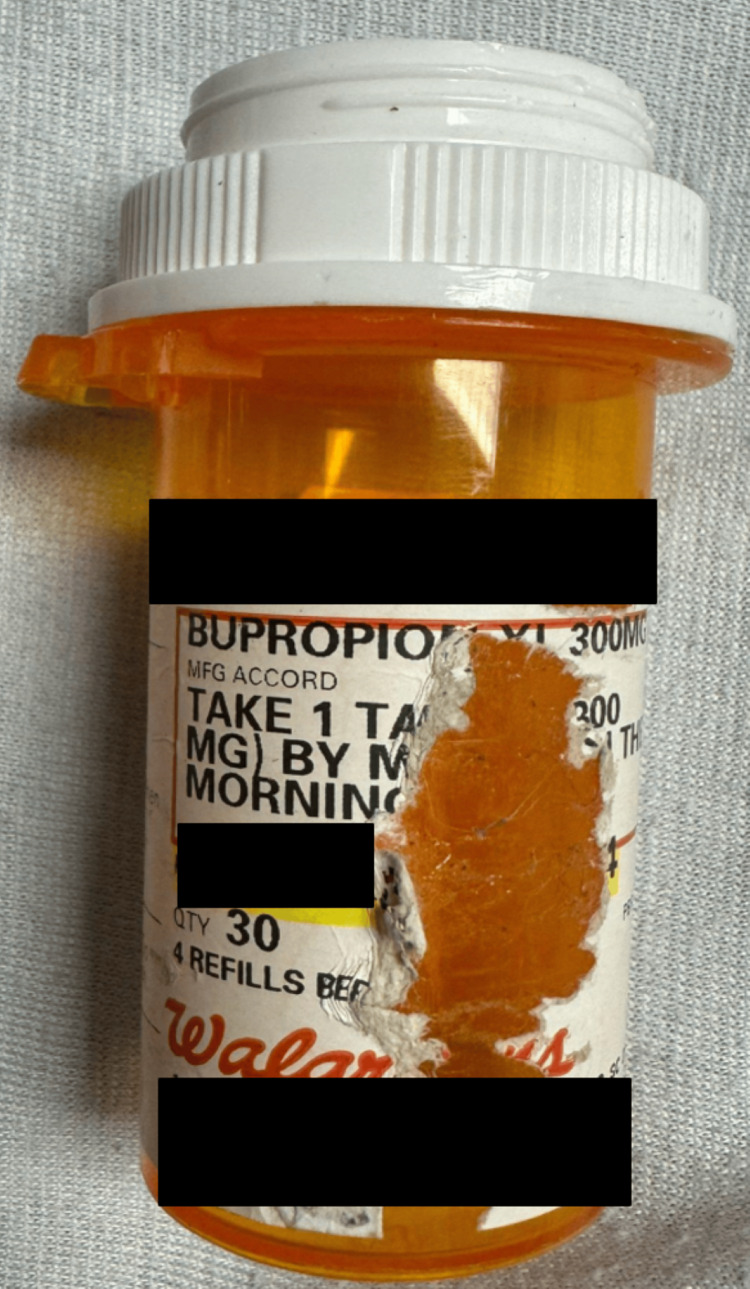
A pill bottle found in the patient's home.

The patient remained stable on day 2 of admission. Further conversation with the mother revealed the child likely accessed a medication a family member takes for smoking cessation, and a home search yielded an empty bottle of 300 mg bupropion tablets in a kitchen drawer that previously contained an unknown amount of tablets, as shown in Figure 3.

**Figure 4:**
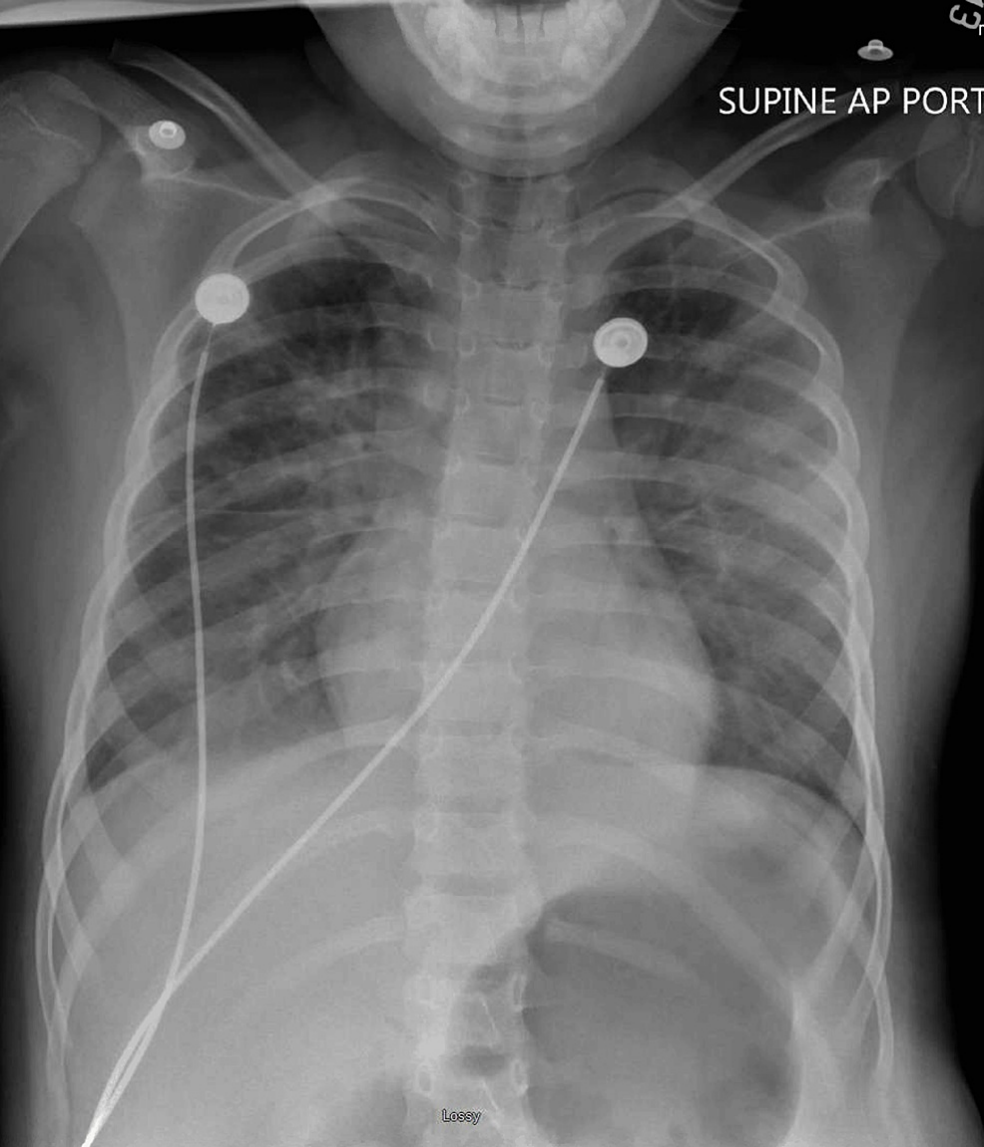
The patient's chest X-ray on day 2 of admission.

The patient's vitals remained stable, and serial EKGs showed a return to normal sinus rhythm. Given the patient's improving clinical picture, a suspected causative agent of the seizure activity, and a benign physical exam, it was decided to extubate the patient 25 hours after intubation and initiate a high-flow nasal cannula (HFNC). A repeat X-ray 12 hours post-extubation later showed improved lung inflation bilaterally, but progressing and increased bilateral perihilar hazy, reticular opacities, as shown in Figure 4 below.

The patient began to decline unexpectedly in the early hours on day 3 of admission. On physical examination, the patient was ill-appearing, with intercostal retractions, decreased breath sounds bilaterally, and a rigid abdomen and extremities with sporadic tremors and hyperreflexia. Patient vitals revealed an HR ranging from 129 to 138 bpm, an RR 36-50 rpm, and a SpO2 of 86-92%, with sporadic desaturations into the 60-70% range. Axillary temperatures showed the patient was febrile with temperature peaking at 100.8°F. Subsequent labs showed an elevated white blood cell (WBC) count of 17.8x10^3^/uL (normal: 4.8x10^3^-10.8x10^3^/uL), C-reactive protein (CRP) of 8.6 mg/dL (normal: <0.3mg/dL), creatine kinase (CK) of 783 IU/L (normal: <250 IU/L), and myocardial band creatinine kinase (CKMB) of 17.60 ng/mL (normal: 0.10-6.00 ng/mL). Lactic acid, troponin I, capillary blood gas labs, and blood cultures yielded levels within normal ranges.

The patient's exposure to bupropion, a serotonergic agent, fevers, and presence of tremors plus hyperreflexia fulfilled the Hunter criteria for serotonin syndrome, and supportive care was initiated per standard of care.

**Figure 5:**
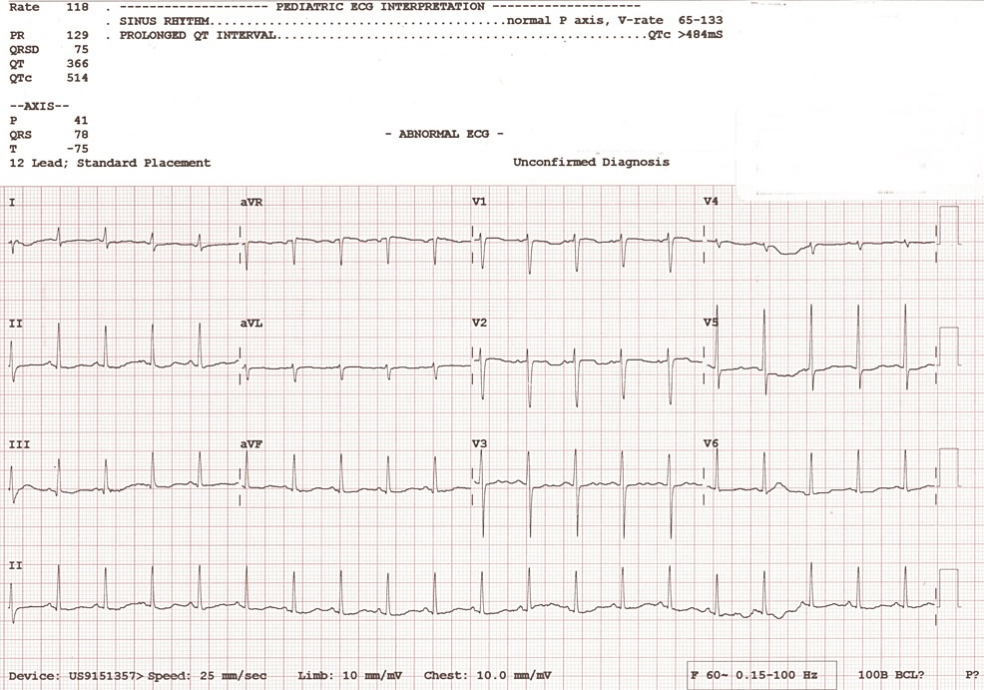
The patient's EKG showed prolonged QTc on day 3 of admission. EKG: electrocardiogram

**Figure 6:**
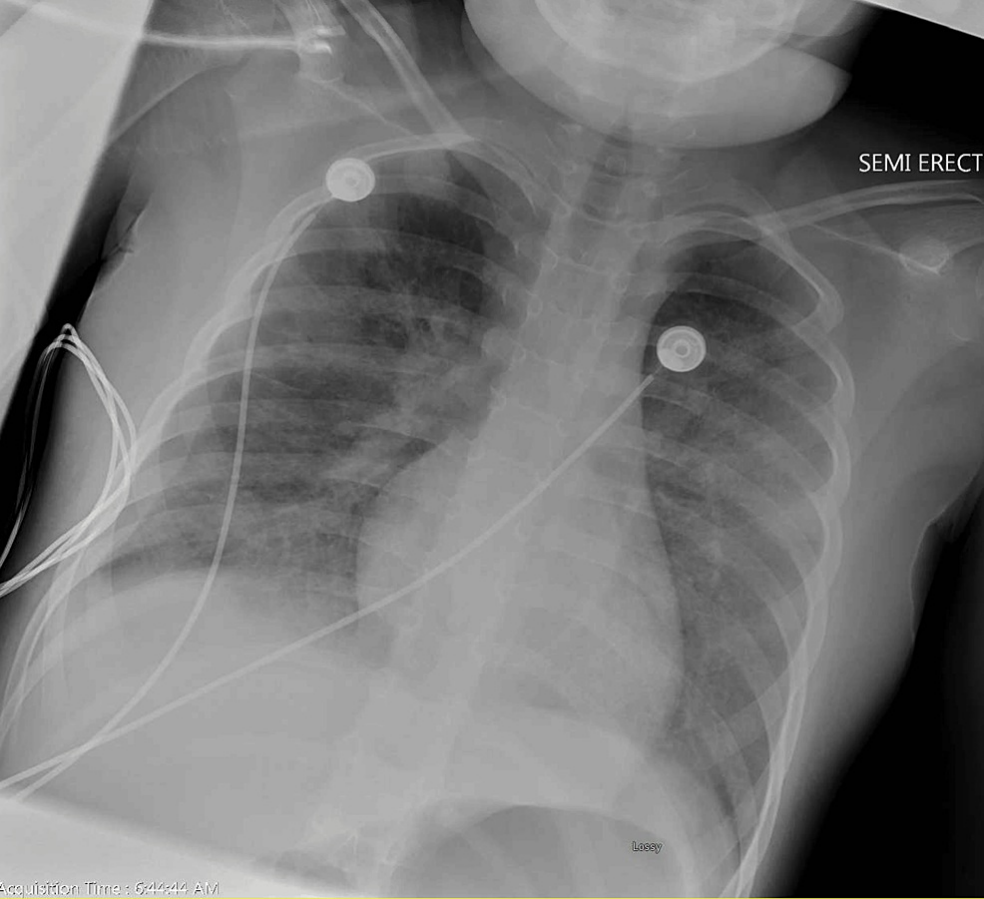
The patient's chest X-ray showed signs concerning for pulmonary edema.

Following intervention, the patient's vitals and physical appearance improved, though she continued to have mild increased work of breathing with retractions. A subsequent EKG showed prolonged QTc of 514 milliseconds (normal: QTc <460 milliseconds) as shown below in Figure 5. A chest X-ray showed persistent opacities in both lower lung fields, more prominent and partially obscuring the right hemidiaphragm, concerning for pulmonary edema, as shown in Figure 6. 

**Figure 7:**
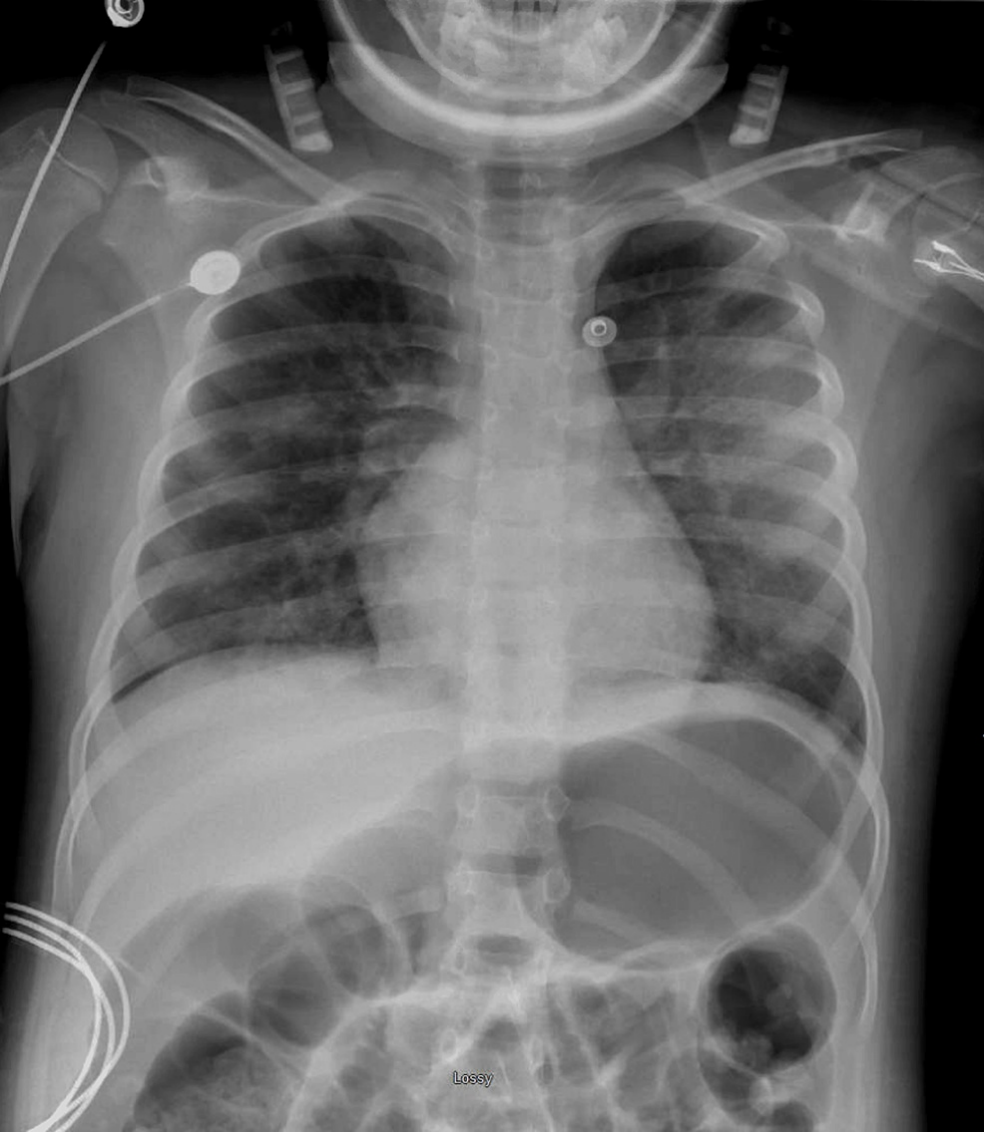
The patient's chest X-ray 10 hours later following BiPAP showed resolving pulmonary edema.

BiPAP was initiated and IV magnesium sulfate was given at a dose of 50 mg/kg for the prolonged QTc. The patient improved clinically over the next 18 hours, and a repeated chest X-ray 10 hours later showed no pleural effusion or pulmonary edema, as shown in Figure 7.

The patient showed remarkable improvement on day 4 of admission. Sedation was weaned in the early morning hours, and the patient transitioned from BiPAP to HFNC, which concluded four hours later, and then to room air. Subsequent EKGs showed resolution of the prolonged QTc. On days 4 and 5 of admission, lab values showed reduced inflammatory and muscle breakdown markers, including a WBC of 13.2x10^3^/uL, a CK of 422 IU/L and 248 IU/L, and a CKMB of 10.90 ng/mL and 7.60 ng/mL. Vital signs remained stable overnight, and the patient was discharged five days after admission with resolution of seizure activity, pulmonary edema, prolonged QTc, and serotonin syndrome.

## Discussion

Bupropion is an antidepressant medication thought to inhibit norepinephrine and dopamine reuptake. Typical side effects include nausea, insomnia, and nervousness, and it is contraindicated in patients with a history of seizures or eating disorders. Bupropion overdoses can present with a variety of symptoms, including seizures, tachycardia, and EKG abnormalities as seen in our patient. Management of bupropion overdoses is largely focused on keeping the patient stable and treating subsequent manifestations. Due to bupropion's chemical structure, it can cause false-positive amphetamine screening, as seen in this case.

In this patient, we report a case of pediatric bupropion overdose with typical factors, including seizures, tachycardia, and prolonged QTc, as well as atypical features, including a delayed presentation of serotonin syndrome and pulmonary edema.

Enterochromaffin cells produce serotonin, which plays a systemic role in motility, vasoconstriction, and bronchoconstriction [5]. Serotonin syndrome/toxicity typically manifests as diaphoresis, tachycardia, hyperthermia, hypertension, tremor, myoclonus, or hyperreflexia within 24 hours of ingestion. Interestingly, our patient did not present with these symptoms until 36-48 hours after ingestion. Serotonin syndrome has been implicated in bupropion overdoses, albeit often involving a concomitant SSRI, SNRI, monoamine oxidase inhibitor (MAO-I), or tricyclic antidepressant (TCA) [5]. Toxicity from isolated bupropion overdoses has been identified in the case of a 14-year-old male and a 19-year-old female and a retrospective study which identified six cases of serotonin toxicity with a median dose of 2,250 mg [12,13]. Unfortunately, the dose in this case is unknown, and bupropion serum concentration levels were unable to be processed. Of note, serotonin toxicity is thought to be an underreported phenomenon as it is a clinical diagnosis assessed by Hunter criteria or if a serotonergic agent is present [14].

Noticeably, the non-cardiogenic pulmonary edema in this patient's hospitalization is atypical for a bupropion overdose. While a marker of cardiac muscle breakdown, the elevated CK-MB was likely elevated due to skeletal muscle breakdown secondary to serotonin toxicity as the CK-MB Relative Index (CK-MB RI = CK-MB (ng/mL) × 100/CK (IU/L)) was 2.24%, less than 3%, likely indicating skeletal source, versus cardiac sources indicated with values >5% [15]. A case in France describes a 35-year-old male in profound status epilepticus as well but with signs and imaging consistent with cardiogenic shock and pulmonary edema [8]. Aside from the prolonged QTc, our patient had no other cardiac abnormalities or clinical signs indicative of cardiogenic pulmonary edema. Non-cardiogenic pulmonary edema can be caused by acute respiratory distress syndrome (ARDS) due to acute viral infection and, less commonly, high altitude, neurogenic, re-expansion, opioid overdose, and other causes, many of which have poorly understood mechanisms. Grand mal seizures have been implicated in neurogenic pulmonary edema and therefore may have played a role [16]. Like serotonin syndrome, the delayed nature of the pulmonary edema's presentation relative to the bupropion overdose is unique. As seizures are a concern with bupropion overdoses, monitoring for signs of neurogenic pulmonary edema may be important for clinicians moving forward.

## Conclusions

Bupropion overdose can lead to severe, life-threatening complications including seizures, cardiac abnormalities, and serotonin syndrome. Applying treatments for complications of bupropion overdose may be complicated by the delayed presentation of symptoms relative to the time and dose of the exposure. Awareness and management of uncommon complications of bupropion overdose may be key to reducing mortality.
